# Communicable Diseases of Domestic Animals That Materially Affect the Livestock Industry1Read before the Tennessee Agricultural Association, January 4, 1900.

**Published:** 1900-03

**Authors:** W. C. Rayen

**Affiliations:** Nashville, Tenn.


### COMMUNICABLE DISEASES OF DOMESTIC ANIMALS THAT MATERIALLY AFFECT THE LIVESTOCK INDUSTRY.[1]

By W. C. Rayen, M.D.C.,
NASHVILLE, TENN.

The time has come when communicable diseases among domestic animals in any community is a subject of paramount importance to livestock raisers and to those who have charge of the various sanitary boards for the protection of the public health. The com-

[1] Read before the Tennessee Agricultural Association, January 4, 1900.

municable diseases of domestic animals are not only a source of great loss to the agricultural classes through the death of large numbers of animals, but where a specific disease affecting animals exists permanently it becomes necessary for those in charge of sanitary work to make stringent rules for the movement of animals from one section to another.

The mere fact that such methods become necessary in any community to protect other sections from having their herds infected at all times places a suspicion on such animals, and they will rarely ever bring the full market price. Where the movement of such animals is attempted (which can only be done for a definite purpose), the car in which they are shipped is branded "from an infected district," and when they are unloaded at the point of destination they are placed in pens branded in the same manner, and can be sold but for one purpose, thus placing the owner at a disadvantage with persons who offer animals from sections free from infection, that can be purchased with confidence.

By far the most important are the diseases affecting domestic animals that can be transmitted to the human family through the meat-supply and milk-supply, thereby communicating some of the most deadly diseases that affect mankind. It is now accepted as a positive fact that human and bovine tuberculosis (consumption) are identical, and can be transmitted to persons through the meat and milk; while other diseases render the meat of animals so affected unfit for human food. As an evidence that such conditions do exist, and that the dangers are not overdrawn, we point to the admirable system of meat-inspection inaugurated throughout the country by the United States Department of Agriculture, under the direction of the Bureau of Animal Industry, for the inspection of all meats intended for foreign shipment and for interstate trade; to the numerous municipal inspections inaugurated throughout the country for the inspection of animals slaughtered for local consumption. All such inspection will be found in charge of persons who are educated and specially trained for such work, thus assuring the purchasing public of the purity of the meat they buy. Under such circumstances it becomes necessary that every effort should be made to eradicate all communicable diseases that affect domestic animals, and to see that the infection of other sections is not brought to and communicated to our own herds. Our domestic animals are from time to time affected with the same diseases that affect animals in other sections, and I shall briefly notice a few of the more important affecting food-producing animals.

*Tuberculosis.* In this State, as far as can be ascertained, tuberculosis is confined among animals to the bovine almost entirely. While there may be some isolated cases in the rural districts, what we have is mostly confined to cattle in and around the cities and large towns. While there has never been an extended investigation of the subject, from what few tests with tuberculin that have been made by veterinarians of various sections of the State, it would seem that the percentage of animals suffering from this disease is much less than in many other sections of the country. The official reports from some States show that the percentage of tuberculosis among dairy cattle is quite large. We are of the opinion here that if the same facts could be collected the percentage found affected with the disease would be small. In fact, it is entirely possible that all cattle in rural sections would be found free from the disease. The value of such information would be a more extended market in which to dispose of surplus milch cows. Then, if it were possible for us to announce officially that our breeding herds were free from tuberculosis, can there be any doubt about the increased value of such animals?

In the States where the percentage of tuberculosis was found to be greatest, there the most determined effort is being made to eradicate the disease. Large numbers of reacting cattle are constantly being destroyed, thus making a market at full prices for sound dairy animals. The tuberculin-test as a means of diagnosis has given us a most reliable agent for the detection of the disease where a physical examination fails to reveal its presence. Where its use has been most extended the prejudice formerly entertained by many persons has gradually become dispelled, and in many sections where the disease is known to exist voluntary application is made to the authorities for the testing of herds. Where the subject is made most clear to the breeding public, there the pressing question is not, Shall tuberculosis among cattle be suppressed? but is, How shall tuberculosis among cattle be suppressed?

Up to the present time much has been accomplished in various sections with the view of eradicating the disease; many different methods of procedure have been adopted. At present what is known as the Danish system, under the leadership of Professor Bang, in a more or less modified form, seems to prevail in sections where the most practical results are being obtained; under this system, after herds have been tested, the reacting animals are separated, the premises are disinfected, the progeny of reacting mothers are separated at birth and raised either on pasteurized milk or milk from a cow that is known to be sound; animals in the most advanced stages of the disease are destroyed, and the carcass is made into fertilizer.

A system similar to this has been pursued by the State Livestock Sanitary Board of Pennsylvania, where up to the present time 33,147 cattle have been investigated and tested, of which 4561, or 13.8 per cent., were tuberculous. They report the most gratifying results in eradicating the disease where the herds were thoroughly infected, and conclude with the following question: "Since tuberculosis has been exterminated on so many farms and in so many districts which were constantly growing more numerous, and since the inflow of tuberculous cattle has been cut off, are we not justified in looking forward to the time when the losses from this disease will be reduced to insignificant proportions?"

The States of Pennsylvania, Illinois, and Kansas now have direct statutes prohibiting dairy-cattle from being brought into the State without a certificate of the tuberculin-test, and most of the New England States regulate the entrance of dairy cattle by the rules of the various livestock sanitary boards. The fact that we are not at the present time seriously affected with this disease is no reason why we should not take every possible precaution to repress its further advance. The statistics of all countries have shown that where the disease once exists its progress is certain unless positive methods are adopted for its extermination.

*Texas Fever.* It is in this disease, of all others, that in various ways the agricultural population of this State are most interested, and from which the annual loss, in the opinion of those who have given the subject the most attention, has never been approximately estimated. As is well known, the direct loss by death alone is enormous, and when it is considered that the market value of all cattle is more or less affected, from the fact that a considerable district within the State is regarded as permanently affected with the disease, it can be plainly seen that the entire loss becomes exceedingly great. Then, again, our condition is such that there is very little encouragement for farmers to bring in other and larger animals for the improvement of our herds, through which cause great gain might be made in the amount we receive from the production of beef animals. The present supply of beef-producing cattle throughout the State is largely of no particular variety, is usually small in size, and mostly originates in the vast range lands of the States South; they have in the past been driven North during what is known as the "open season." These cattle require more time and more feed for their development than cattle of the more prominent beef-producing varieties. Large numbers of these cattle come from the section that may be truly termed the permanently infected district of Texas fever, for they invariably carry the microörganism in their circulation that produces the disease among susceptible cattle. From these facts, which may be said to be accepted by those who have given the subject the most attention, it becomes imperative that every person who is interested in agriculture should become thoroughly conversant with every feature of the question in order that action may be taken with the view of eventually eradicating the disease from the State.

Much information in regard to Texas fever has already been obtained through the efforts of the United States Department of Agriculture and the various experimental stations throughout the country and by those who have been engaged in field work in the quarantine service. It is shown that the disease is caused by a blood-destroying microörganism. It is known that this microorganism is transmitted from cattle to cattle through the agency of a specific tick (*Boophilus bovis*). There is, however, a question that has never been established by experiments or observation that might have an important bearing on the subject in this State. The primary origin of the microörganism that causes Texas fever among cattle has not been positively located. Considerable evidence has accumulated that would seem to indicate that all ticks (*Boophilis bovis*) do not carry the specific micro-parasite that causes Texas fever, and are therefore incapable of infecting susceptible cattle. Such a fact, if established, would without a doubt very materially modify existing conditions in this State.

*Quarter-evil, Black Quarter, or Black Leg.* This disease, characterized by the development of an extensive inflammatory tumor in some part of the body, has caused great loss among cattle in the States west of the Mississippi River, and has made its appearance in some localities in this State. It is a disease that affects young animals in most cases. The susceptibility of cattle seems to diminish greatly after two years of age. It was at one time a universally accepted opinion that quarter-evil was mainly a disease of forced or overfeeding ; this opinion has been rightly abandoned, and it is now certain that the disease often attacks animals in poor or medium condition ; the disease is caused by a specific microorganism—the bacillus of quarter-evil ; it is a strictly infectious disease and is confined to certain farms and localities. All treatment is doubtful ; prevention is the only course to pursue where the disease makes its appearance; this is accomplished through vaccination with a vaccine now prepared and furnished free of charge to local authorities by the United States Department of Agriculture.

*Hog-cholera* is a widely disseminated disease that annually causes great loss to the livestock industry. The disease has been under investigation for many years, and, while the cause is well understood, no curative remedy has been discovered that is of practical value. It is a disease caused by a specific microörganism, and it is the opinion of prominent authorities that it can be largely controlled through a well-organized system of sanitary science and police, through which swine epizoötics would soon cease to be the cause of serious loss. While it might not be possible to stamp out this disease on account of the conditions that prevail in this State, the loss can be so much reduced as to be of little moment.

The so-called cures so extensively advertised have proved ineffectual in a series of tests made by various experiment stations throughout the country. By what is known as serum-therapy, the United States Department of Agriculture has for the past two or three years been making practical or field experiments, and from the reports the result seems to be most gratifying.

In the management of all communicable diseases among domestic animals the lines of action followed according to the well-defined rules of veterinary sanitary science and police when intelligently administered have at all times obtained the most satisfactory results. Every fatal contagion or infectious disease that affects domestic animals is caused by a specific microörganism that in each case has a life-history of its own, and all measures for the prevention and restriction of such diseases must be based on the characteristics of each particular parasite.

Animals once affected with such diseases rarely ever thrive if recovery should occur. From an economic point of view prevention and restriction through the application of rational methods is the intelligent course to pursue. The public health demands the purity of animals food-products, and the public wealth requires that animals should be kept free from the communicable diseases that so materially affect the profits obtained through the livestock industry.

